# Spinopelvic Relationship and Its Impact on Total Hip Arthroplasty

**DOI:** 10.1016/j.artd.2022.07.001

**Published:** 2022-08-19

**Authors:** Stefan Louette, Alice Wignall, Hemant Pandit

**Affiliations:** Trauma and Orthopaedics, Leeds Teaching Hospital Trust, Leeds, UK

**Keywords:** Total hip arthroplasty, Pelvic tilt, Spinopelvic mobility, Dislocation, Navigation systems

## Abstract

The dynamic, complex interaction among the spine, pelvis, and hip is often underappreciated, yet understanding it is vital for both arthroplasty and spinal surgeons. There is an increasing incidence of degenerative hip and spinal pathologies as a result of the ageing population. Furthermore, hip pathology can cause spine pathology and vice versa through “hip-spine” and “spine-hip syndrome.” Consequently, total hip arthroplasty (THA) and spinal fusion surgery, which both affect spinopelvic mobility, are also on the rise. Alteration in spinopelvic motion can affect the orientation of the acetabulum and, therefore, implant positioning in THA, leading to complications such as dislocation, impingement, aseptic loosening, and wear of components. This makes it imperative to assess spinopelvic motion and pelvic tilt prior to patients undergoing THA. In this paper, we explore how the surgeon should proceed to reduce risk of component malalignment, as well as the role of navigation systems in acetabular cup positioning.

## Introduction

Upright posture and bipedal gait put considerable and unique demands on the human body [1]. The spine and pelvis work together in “biomechanical concert,” an effect often underappreciated by clinicians [2]. When moving from a standing to a sitting position, the pelvis, spine, and hip change their relative position to allow movement of the femur and hip flexion while maintaining the line of gravity close to the acetabulum’s centre. The majority of movement (∼75%) occurs at the hip, but there is also tilting of the pelvis (also known as pelvic tilt [PT]) and flexion of the lumbar spine [3]. PT being defined as the rotation of the pelvis around the horizontal axis (coronal plane).

Both hip and spinal pathologies reduce range of movement, which in turn impacts the movement occurring at the spinopelvic junction. In these cases, the spine and hip do not work in harmony and cause excessive and/or abnormal movement. Due to the altered biomechanics, spine pathology can lead to hip pathology and vice versa. This is termed “spine-hip syndrome” when the spine has the initial pathology, or “hip-spine syndrome” if it is the hip. Either can lead to persistent symptoms and higher complication rates after a surgical intervention to treat them [4,5].

US data show that 2% of all primary total hip arthroplasty (THA) patients will have had lumbar spinal fusion (LSF) prior to THA [6]. History of spinal fusion has been identified as the strongest predictor of dislocation in the first 6 months after surgery [7]. This has been linked to the aforementioned adjustments in movements at the spinopelvic junction causing alterations to PT in these patients. Seventy percent of revision THAs for dislocation and 87.5% of late dislocations can be linked to spinopelvic imbalance [2].

Mechanical complications of THA such as impingement, dislocation, aseptic loosening, and wear result from component malpositioning. Optimal intraoperative component orientation requires a detailed assessment of the spinopelvic relationship, as the true pelvic position cannot accurately be determined intraoperatively by the naked eye. Computer navigation can be helpful yet needs to take into account the functional cup position rather than just the anatomical and intraoperative positions. These systems provide the surgeon with real-time feedback to allow for a more-precise cup position; however, at present, their costs are still unproven [8].

This paper aims to provide a synopsis of the current understanding of the spinopelvic relationship and its impact on PT with reference to THA, as well as to identify methods to reduce risk of component malalignment.

### What is the impact of changing body position?

PT changes with body position even in healthy individuals [2]. In the standing position, there is an anterior PT. This, when combined with physiological lumbar lordosis, allows the acetabulum to position itself to cover the femoral head, permitting the hip extension needed for mobilizing. However, when transitioning to the sitting position, the pelvis tilts posteriorly (∼20°) anteverting the acetabulum (by 15° to 20°). This transition allows hip flexion without anterior impingement or posterior dislocation, resulting in a more-efficient movement of the femur [8].

### Other factors contributing to PT

Greater anterior PT and acetabular anteversion have been demonstrated in women than in men [[9], [10], [11]]. With increasing age, posterior PT increases. Ageing is linked to disc dehydration and reduced lumbar lordosis, which in turn causes pelvic retroversion. Hip extension becomes increasingly pronounced, leading to compensatory posterior PT [12]. Pregnancy leads to an increased anterior PT, particularly during weeks 12-36; postpartum, the pelvis begins to return to prepregnancy configuration, but currently no evidence exists about how long this persists [13].

### Impact of hip and spinal pathology on spinopelvic movement and PT

The Bordeaux classification attempts to classify abnormalities of the spino-hip relationship with 2 terms, “spine-hip syndrome” and “hip-spine syndrome”, depending on the joint where the abnormality originates, hip or spine [14].

#### Spine-hip syndrome

When standing, patients with flat backs were more posteriorly tilted than “balanced” patients [15]. Greater flat-back deformities correlated with a significantly higher anterior PT change when patients change stance. Scoliosis leads to a compensatory alteration in PT, with a posterior PT recorded in the standing position [16]. Lumbar degenerative disease has been linked with a posterior PT while standing but a more-anterior PT when sitting [17]. This is a result of an altered sitting mechanism in spinal pathology cases with spinal flexion substituted for hip flexion. Lumbo-sacral fractures may also change the PT, even when healed [18]. Spinopelvic motion has been categorized into 4 different types depending on lumbar spine pathology by Phan et al. [19] (Table 1). Flexibility looks at the lumbar spine, whereas balance uses a C7 plumb line [19].

**Table 1:** Phan classification of spinopelvic motion.

Classification	Found in
Balanced and flexible	Healthy population
Balanced and stiff	Lumbar degenerative disease, prior LSF
Unbalanced and flexible	Post-laminectomy, neuromuscular kyphosis
Unbalanced and stiff	Long LSF, ankylosing spondylitis

#### Hip-spine syndrome

Acetabular dysplasia is a common cause of hip-spine syndrome. These patients have an anterior acetabular coverage defect resulting in anterior PT to avoid “edge-loading” [20,21]. Primary hip osteoarthritis is an important cause of hip-spine syndrome. Osteophytes and capsule contracture lead to reduced hip mobility and flexibility [9]. This leads to increased lumbar lordosis, a greater role for the spine when transitioning position, and an anterior PT [22]. Hip osteoarthritis has also been shown to lead a substantially greater change in PT when transitioning from standing to walking [23].

### Impact of THA on PT

Several studies demonstrate no change between preoperative and postoperative PT [24,25]. Kanto et al. found that ∼60% of patients had no change at 1 year following THA, with over 81% having <10° change [26]. Pelvic retroversion was more common than anteversion (25% vs 16%, respectively) although this was not statistically significant. Preoperative altered PT was the only predictive factor associated with a marked change in postoperative PT. Anterior PT preoperatively had a significant postoperative posterior PT, and vice versa [27]. However, this is not always the finding. Ishida et al. observed PT change in patients with pre-existing anterior PT, but not for those with a posterior PT [28]. But postoperative changes were heavily influenced by age, with younger patients having largely posterior changes and older patients tending to have anterior changes [26].

Taki et al. reported a significant difference in both standing and sitting PT postoperatively at yearly intervals (1-4), with PT changing at all the recorded time sessions [29]. Age, female gender, and alteration in PT prior to operation were found to be the greater contributors to postoperative changes [28].

### Impact of LSF on PT

Matsumoto et al. assessed PT in patients after lumbar fusion for scoliosis and found that 73% with reduced lumbar lordosis displayed an increased posterior PT [29,30]. Longer spinal fusions and spinopelvic fusions can alter the sacral slope during postural transitioning, with a decrease of 0.9° anteversion for each additional level of spinal fusion. Nam et al. found that patients with a history of lumbar or lumbosacral fusion had a more posterior PT in the standing position but a more anterior PT in the seated position, thereby implying a lack of compensatory PT when shifting position [30].

### Impact of LSF on THA

Two meta-analyses have been performed looking at THA and LSF outcomes. An et al. found LSF to be a significant risk factor for increased dislocation rates (relative risk 2.03; *P* < .00001) and need for revision (relative risk 3.36; *P* = .006) [31]. Patient-reported outcomes were also worse in these patients. However, a meta-analysis for this could not be performed due to nonhomogeneity [31]. Wyatt et al. echoed these findings, reporting that patients with LSF have “a substantially and significantly increased risk” of dislocation and revision but also that there was significantly increased risk of aseptic loosening, periprosthetic fracture, joint infections, and other adverse events [32]. This was true in patients with long as well as short spinal fusion [32]. Table 2 provides a summary of the literature.

**Table 2:** Summary table of the literature comparing outcomes of THA with or without prior LSF.

Study	Design	Number	Outcomes of THA with prior LSF (comparator group, those without prior LSF)
Sing et al., 2016 [12]	Retrospective cohort	598,995	LSF led to higher rates of dislocation, revision, loosening, and any prosthetic-related complication within 24 mo (*P* < .001)
Barry et al., 2017 [33]	Retrospective cohort	105	LSF led to higher rates of complications (31.4% vs 8.6%, *P* = .008), reoperation (14.3% vs 2.9%, *P* = .040), and general anaesthesia (54.3% vs 5.7%, *P* = .0001). Long LSF (>3 levels) led to increased postop analgesia consumption (*P* = .001)
Perfetti et al., 2017 [34]	Retrospective case-control	934	LSF led to higher rates of dislocation (RR = 7.19; *P* < .001) and revision rates (RR = 4.64; *P* < .001)
Diebo et al., 2018 [35]	Retrospective cohort	49,920	LSF led to increased hip dislocation (OR = 2.2 [*P* = .002] [short, 2-3 levels] and 4.4 [*P* < .001] [long >4 levels]). Increased revision rates (OR = 2.0 [*P* < .001] [short] and 3.2 [*P* < .001] [long])
York et al., 2018 [36]	Retrospective cohort	460	LSF led to a higher dislocation risk (RR = 4.77; *P* ≤ .0001), and dislocators with LSF had higher revision rates (RR = 3.24; *P* = .003)
Malkani et al., 2018 [37]	Retrospective cohort	62,387	LSF led to more dislocation (prevalence = 7.4% vs 4.8% in control; *P* < .001). LSF led to 48% higher revision rates.
Parilla et al., 2019 [38]	Retrospective cohort	292	LSF increased dislocation risk (RR = 3.0) and revision (RR = 2.7)
Buckland et al., 2017 [39]	Retrospective cohort	14,747	LSF led to higher rates of dislocation: 1 to 2 levels of fusion (OR = 1.93; *P* < .001), 3 to 7 levels (OR = 2.77, *P* < .001)
Gausden et al., 2018 [7]	Retrospective cohort	207,285	LSF was highest independent predictor of dislocation (OR = −2.45; *P* < .0001)
Salib et al., 2019 [4]	Retrospective cohort	84	LSF with sacrum involvement increased dislocation risk (HR = 4.5; *P* = .03)
Furuhashi et al., 2021 [40]	Retrospective cohort	23	LSF had a dislocation rate of 22%
Lazennec et al., 2017 [41]	Retrospective case-control	243	LSF led to reduced adaptability of the lumbosacral junction with significant alterations to PT
Eneqvist et al., 2017 [42]	Retrospective case-control	997	LSF led to worse PROMs at 1 y postop
Loh et al., 2017 [43]	Prospective cohort	164	LSF led to worse PROMs at 6 mo (*P* = 0 .046) and 2 y (*P* = .054)
Grammatopoulos et al., 2019 [44]	Retrospective case-control	42	LSF led to inferior PROMs (*P* < .001), more surgery-related complications (loosening, periprosthetic fracture or infection, psoas irritation; *P* = .013), and dislocation (*P* = .023)

HR, hazard risk; LSF, lumbar spinal fusion; OR, odds ratio; PROMs, patient-reported outcomes; PT, pelvic tilt; RR, relative risk.

### How should the surgeon proceed?

Zhu et al. found that 95% of patients undergoing THA had a degree of anterior or posterior PT, with 16% having >10° tilt [45,46]. Data show 18%-25% of patients undergoing THA have spinal pathology for which they have seen a spinal surgeon prior, which as previously described will result in PT changes and increased risk of complications [46]. It is therefore imperative to try to identify modifications that can be performed preoperatively, intraoperatively, and postoperatively to improve complication rates in a sizable number of high-risk patients. In addition, patients should be informed during consenting that existing spinal fusion means they are a high-risk group for dislocation, revision, and complications [12].

### Preoperative planning and assessment

Yang et al. and Mancino et al. recommend that prior to THA, all patients must undergo standing, supine, and sitting lateral radiographs of the pelvis and the lumbar spine [47,48]. The views should ideally include L1 or, at the least, the level of L3, as most of the lumbar motion happens between L3 and L5 [47,48].

Most of the radiographic analysis of the hip is undertaken on the “standard” anteroposterior (AP) view radiograph, which has the acetabulum in the coronal plane, [10] as a standing film will more accurately represent the functional pelvis position than supine radiographs [49]. However, safe position in the sagittal plane may be more important in patients with existing spinal fusion. It has therefore been recommended that 3 views of the pelvis should be obtained: lateral standing, sitting, and AP standing [10]. Imaging assessing movement while changing stance preoperatively has also been recommended [24].

### THA or spinal fusion first?

Sultan et al. originally argued that the most-troublesome issue should be managed first while monitoring the other [23]. However, their recommendation makes an exemption in the presence of hip flexion contracture, which may clinically mimic or worsen symptoms of spine deformity. If present, it has been advised to perform THA first, as (1) hip flexion contractures and spinal deformity often improve with THA and (2), for best outcomes with spinal fusion, it is important hip flexion contractures have been dealt with before [23]. Various authors have reported different outcomes based on the order of surgery, from no significant differences in revision and instability [37,43] to decreased dislocation and revision rates when THA is performed before LSF [6,50] and to the opposite with prior THA leading to significantly increased rates of dislocation, infection, revision, and postoperative opioid usage [51]. Unfortunately, all these studies are limited by not evaluating the relevance of timing between operations and by being retrospective case-control studies.

One specific group has been flagged as benefiting from undergoing spinal fusion first: patients with excessive pelvic retroversion due to their spine pathology, for example, patients with ankylosing spondylitis. Hu et al. found that a spinal osteotomy in these patients led to correction of their acetabular abduction and anteversion, thereby allowing relatively normal acetabular orientation and a hypothetical decrease in risk of dislocation [50,52]. If LSF is to occur prior to THA, Haffer et al. advise that spine surgeons should be aware of a hip flexion contracture and should warn the patients of an increased risk of complications with existing or planned THA [51,53].

### Acetabular cup placement

The orientation of an acetabulum or an acetabular prosthesis is traditionally described by its inclination and anteversion. Orientation can be assessed anatomically, radiographically, and by direct observation at operation. The angles of inclination and anteversion determined by these 3 methods differ because they have different spatial arrangements. There are therefore 3 distinct definitions of inclination and anteversion.

In 1993, Murray highlighted the fact that operative anteversion is measured around a transverse axis, anatomical anteversion around a longitudinal axis, and radiographic anteversion around an oblique axis [52]. The author also developed nomograms to allow conversion of one orientation to the other two. Murray concluded that operative definitions be used to describe the prostheses orientation while anatomical definitions be used for normal/dysplastic acetabula. If the orientation is determined from an AP radiograph, it should be converted to operative orientation before being quoted. Anatomical anteversion is best determined from computed tomography (CT) or magnetic resonance images, as it is measured in the transverse plane [54].

Lewinnek et al. defined a safe zone to minimize dislocation risk, which comprises an operative cup inclination of 40° ± 10° and operative cup anteversion of 15° ± 10° [24,53]. Although considered a useful target, the value of this safe zone has nonetheless been called into question in recent years. In an assessment of 9784 primary THAs performed by high-volume surgeons, Abdel et al. reported that 58% (120 of 206) of those that dislocated were within the Lewinnek “safe zone” [54,55]. This finding is likely due to the multifactorial causes contributing to instability, as well as confusion between anatomic and radiographic cup orientation. In addition, altered PT plays a role. Posterior PT has been shown to increase acetabular component anteversion when standing, which is linked to decreased accuracy of placement within the safe zone from 82% to 64% [26,56,57]. Therefore, the cup positioning defined only intraoperatively may not be ideal for all patients. The categorizations of spinopelvic motion proposed by Phan et al. may be of value here to help surgeons (Table 3) [18,19].

**Table 3:** Summary of alterations to Lewinnek safe zone depending on Phan classification.

Classification	Found in	Alteration to Lewinnek safe zone
Balanced and flexible	Healthy population	Use as described
Balanced and stiff	Lumbar degenerative disease, prior LSF	Increase anteversion (15°-25°)
Unbalanced and flexible	Postlaminectomy, neuromuscular kyphosis	Reduced anteversion
Unbalanced and stiff	Long LSF, ankylosing spondylitis	Reduced anteversion

**Figure 1:**
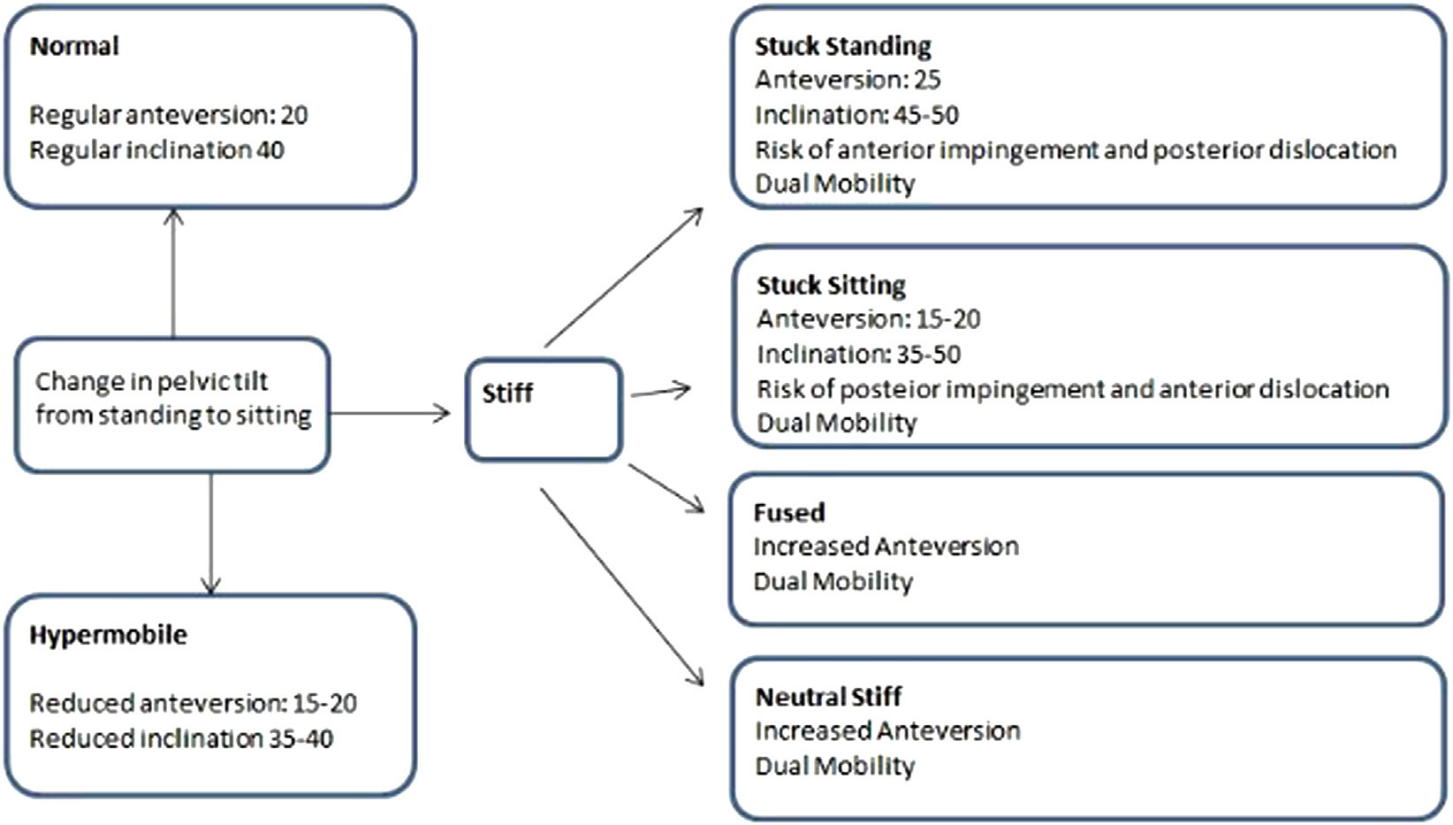
Recommendation of cup placement algorithm, based on Stefl et al. [56].

Stefl et al. describe a further classification system, with 6 classes: normal, stiff (further subdivided into fixed anterior tilt and fixed posterior tilt; PT is present in both sitting and standing), kyphotic, fused, and hypermobile [56,58]. They advise placement as shown in Figure 1.

These classifications can be useful as general categories, but it has been advised that the degree of stiffness and sagittal imbalance should be determined on a case-by-case basis. Increased acetabular inclination is not a “free” solution, as it is a recognized risk factor for accelerated bearing surface wear and linear fractures [59]. Additionally, it should be noted that as patients age or if their spine disease progresses, they may transition into a category that may increase the risk of late dislocations. In contrast, THA may increase spinopelvic mobility. Stefl et al. found that 54% of patients undergoing THA had normal spinopelvic mobility preoperative and that this increased to 80% after THA, which has been attributed to intraoperative release of hip flexion contracture [9,56,58].

### Hip component

Dual-mobility cups have been shown to decrease the risk of instability in high-risk patients, both in primary and revision THA, and it is unsurprising they have been mentioned when thinking about THA in patients with altered PTs [51,60]. Tezuka et al. and Nessler et al. looked at dual-mobility cups in patients with LSF and found reduced dislocation rates [59,60]. Vigdorchik et al. has proposed a new risk-prediction model based on radiological features to try to identify patients who may benefit most from dual-mobility cups. When using this algorithm, there was a significant decrease in dislocation rates (0.5% vs 3.1%) [61].

High-offset stems also seem to have a role in patients with spinal pathology. A study looking at 12,365 patients who underwent THA found that high-offset stems were protective for dislocation (*P* < .0001). While high-offset stems can lead to complications such as bursitis, Vigdorchik et al. did find them to be protective and concluded there was benefit in their usage in patients at high risk to mitigate risk of dislocation [61].

### Role of computer navigation

The pelvis moves when a person alters their position, and the relative change in PT that occurs as a result cannot be accurately assessed by 1 static 2-dimensional AP radiograph of the pelvis. Two-dimensional radiographic images suffer from “out-of-plane” rotations, including pelvis rotation (1° of pelvic rotation can cause 0.8° change in the measurement of acetabular version), femoral rotation, and/or flexion or hyperextension. CT has been proposed as a solution, but the supine position required for imaging does not provide a realistic evaluation of the patient’s compensation mechanisms during weight-bearing [62].

Several computer navigation systems are available that may address the challenge of accounting for dynamic spinopelvic movement during imaging of hips. Computer navigation systems are defined by their shared goal of providing guidance to surgeons on patient anatomy in preoperative planning and intraoperative placement of instruments and implants.

There is support for the notion that navigation improves cup orientation. A meta-analysis by Xu et al. linked computer navigation systems with improvements to the precision of acetabular cup placement (*P* < .00001); however, no significant differences were found in cup inclination, anteversion, or in the incidence of postoperative dislocation [62,63]. Meta-analyses by Liu et al. (2015) and Beckmann et al. (2009) noted that navigation enhanced cup placement and minimized outliers [63,64].

Navigation systems can incorporate a number of techniques developed to correct for PT to better facilitate cup placement. The “kinematic alignment technique” uses the transverse acetabular ligament as a landmark to adjust cup position and judge the patient’s spine-hip relationship. This allows a restoration of the “native” acetabular anteversion and the hip’s centre rotation [65]. Babisch et al. developed a nomogram to allow navigation systems that rely on the pelvic anterior plane to convert cup alignment values [8]. At 1-year follow-up, none of the 98 patients who underwent navigation using this tool sustained a dislocation, and on CT imaging, 99% of cup anteversion and 97% of cup abduction values were in the target range [8]. In analyzing CT data for 420 patients, Haimerl et al. found that the interteardrop and interfossa distances were consistent in pelvises of the same gender, as was the relationship between the anterior pelvic plane and other reference planes reliant on acetabular points [65]. From this, they developed a procedure using intraoperatively available landmarks. Using this tool, they were able to plan THA placement, of which 99% were in the Lewinnek safe zone [65].

In addition to PT, navigation systems can address the multifactorial reasons that can contribute to instability and dislocation, which may improve functional alignment. Clinical data suggest that navigation offers a superior means than conventional methods for achieving the goals of reduced leg length discrepancy (*P* = .004) [62] and offset [[66], [67], [68], [69], [70]].

Robotic-arm-assisted arthroplasty is a similarly novel technique that has been proposed to aid placement of components. When used by a trained professional, robotic-arm-assisted placement was found to be reliable when using bony landmark (83% of cups placed within targets for inclination and anteversion) or using functional planning (90%), with lower variance reported in the functional group [71]. However, Hayashi et al. has found that a posterior PT, as found in patients with spinal pathology, is a predictive factor for inaccurate cup positioning [72].

Although increasing in use, navigation systems and robotic-arm-assisted arthroplasty are still infrequently employed in THA, likely primarily due to concerns around their associated costs and increased surgical time [73].

## Conclusions

It is essential to appreciate the relationship between pelvis, spine, and hips, as well as the impact of pathology on the movement occurring at the spinopelvic junction and in turn on PT. This is particularly important to understand when planning THA, as both spinal pathology and surgery will have an effect on PT and complication rates of THA. Fused spines following an operation or stiff spines from pathology can all affect PT, and in patients with these conditions, the placement of components should be considered. The ordering of spinal/hip surgery, precise cup placement, and type of cup used can all help reduce dislocation rates. Moreover, preoperative THA planning that involves assessing PT and acetabular inclination and anteversion becomes imperative to achieve precise acetabular cup placement. This differs significantly between individuals and is dynamic and varying with different positions and activities. The previously described “safe zones” do not take into account this dynamic behaviour; therefore, accurate cup placement cannot be achieved. Careful preoperative planning of the component alignment on an individual patient basis could improve outcomes and revision rates [72]. Acetabular cup placement is not something that the surgeon can be accurate within a specific range of degrees by themselves. Computer navigation systems and robotic-arm-assisted surgery may aid the surgeon and allow for a more-precise cup position. Ideally, navigation systems should work to address the multifactorial contributors to dislocation and instability, of which spinopelvic factors remain a key but often overlooked element. Postoperative care is also an important element that seems to be forgotten by research with no evidence on success of differing physiotherapy or occupational therapy interventions in at-risk individuals.

## Conflicts of interest

Prof. Hemant Pandit are paid consultants for Medacta International, DePuy Synthes, Smith and Nephew, Meri Life, Invibio, Zimmer Biomet, and JRI Orthopaedics; both receive research support from Medacta International, Zimmer Biomet, DePuy Synthes, and Invibio; and they receive financial or material support from Kennedy’s Law. Mr Stefan Louette and Miss Alice Wignall declares no potential conflicts of interest.

For full disclosure statements refer to https://doi.org/10.1016/j.artd.2022.07.001.

## Appendix A Supplementary Data

Conflict of Interest Statement for Wignall

Conflict of Interest Statement for Pandit

Conflict of Interest Statement for Louette
