# Supplementary material for: Spinopelvic Relationship and Its Impact on Total Hip Arthroplasty
Source: Arthroplast Today. 2022 Aug 19;17:87–93. doi: 10.1016/j.artd.2022.07.001 (PMC9420424; doi:10.1016/j.artd.2022.07.001)
Supplement: Conflict of Interest Statement for Pandit [file mmc2.docx]

# CONFLICT OF INTEREST STATEMENT

***American Association of Hip and Knee Surgeons***

(Adopted from the American Academy of Orthopaedic Surgeons disclosure statement)

The following form **must be filled out completely and submitted by each author (example, 6 authors, 6 forms).**

**All items require a response. If there is no relevant disclosure for a given item, enter "*None*.”**

Manuscript Title: Spinopelvic relationship and its impact on total hip arthroplasty

1. Royalties from a company or supplier (The following conflicts were disclosed) NONE

2. Speakers bureau/paid presentations for a company or supplier (The following conflicts were disclosed) NONE

3A. Paid employee for a company or supplier (The following conflicts were disclosed) NONE

3B. Paid consultant for a company or supplier (The following conflicts were disclosed) Medacta International, Depuy Synthes, Smith and Nephew, Meri Life, Invibio, Zimmer Biomet, JRI Orthopaedics.

3C. Unpaid consultants for a company or supplier (The following conflicts were disclosed) NONE

4. Stock or stock options in a company or supplier (The following conflicts were disclosed) NONE

5. Research support from a company or supplier as a Principal Investigator (The following conflicts were disclosed) Medacta International, Zimmer Biomet, Depuy Synthes, Invibio.

6. Other financial or material support from a company or supplier (The following conflicts were disclosed) Kennedy’s Law

7. Royalties, financial or material support from publishers (The following conflicts were disclosed) NONE

8. Medical/Orthopaedic publications editorial/governing board (The following conflicts were disclosed) NONE

9. Board member/committee appointments for a society (The following conflicts were disclosed) NONE

**Each author must sign AND print or type his/her name, date and submit a separate form**

In addition, one BLINDED Conflict of Interest form (no author names used) should be submitted per manuscript with all author disclosures.

Hemant Pandit
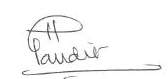
 3^rd^ May 2022

Author Name (Print or Type) Author Signature Date
